# A two-domain folding intermediate of RuBisCO in complex with the GroEL chaperonin

**DOI:** 10.1016/j.ijbiomac.2018.06.120

**Published:** 2018-10-15

**Authors:** Ramanathan Natesh, Daniel K. Clare, George W. Farr, Arthur L. Horwich, Helen R. Saibil

**Affiliations:** a Crystallography and Institute of Structural and Molecular Biology, Birkbeck College London, Malet Street, London, WC1E 7HX, UK; b Department of Genetics, Yale University School of Medicine, Boyer Center, 295 Congress Avenue, New Haven, CT 06510, USA; c Howard Hughes Medical Institute, Yale University School of Medicine, 295 Congress Avenue, New Haven, CT 06510, USA

**Keywords:** RuBisCO, Ribulose-1,5-bisphosphate carboxylase/oxygenase, CO_2_, Carbon dioxide, *E*. *coli*, *Escherichia coli*, GroEL_D473C.His6_, D473C mutant of GroEL with 6 His tag modification on the mutated cysteine 473, KOAc, Potassium Acetate, Mg(OAc)_2_, Magnesium Acetate, DTT, Dithiothreitol, MSA, Multivariate Statistical Analysis, Protein folding, GroEL, Chaperonin, Non-native protein, RuBisCO, Single particle cryo-EM

## Abstract

The chaperonins (GroEL and GroES in *Escherichia coli*) are ubiquitous molecular chaperones that assist a subset of essential substrate proteins to undergo productive folding to the native state. Using single particle cryo EM and image processing we have examined complexes of *E*. *coli* GroEL with the stringently GroE-dependent substrate enzyme RuBisCO from *Rhodospirillum rubrum*. Here we present snapshots of non-native RuBisCO - GroEL complexes. We observe two distinct substrate densities in the binary complex reminiscent of the two-domain structure of the RuBisCO subunit, so that this may represent a captured form of an early folding intermediate. The occupancy of the complex is consistent with the negative cooperativity of GroEL with respect to substrate binding, in accordance with earlier mass spectroscopy studies.

## Highlights

• Cryo EM 3D snapshots of non native *Rhodospirillum rubrum* RuBisCO bound to *E*. *coli* GroEL
• Two-lobed substrate density in the binary complex may represent a captured form of an early folding intermediate
• The occupancy of the complex is consistent with the negative cooperativity of GroEL with respect to substrate binding.
• The RuBisCO density is seen in contact with 3 consecutive GroEL apical domains and one remote apical domain.

## 1 Introduction

Correct protein folding is essential for cell viability in all kingdoms of life and depends on protein quality control systems, in which molecular chaperones play a major role. In animals, protein misfolding and aggregation can produce toxic species that can cause cell death in serious neurodegenerative conditions such as Alzheimer's, Parkinson's and the prion diseases, owing to a failure of chaperones to prevent the accumulation of aggregates [1]. The chaperonin class of molecular chaperones [[2], [3], [4], [5]] was discovered as the *E*. *coli* genes GroEL and GroES (large and small subunit respectively) of the GroE operon required for growth of bacteriophage lambda [6] and the mitochondrial form was identified as a protein folding factor [7, 8]. The *E*. *coli* GroE system was functionally characterised by its role in assisting the folding and assembly of the CO_2_ fixing enzyme ribulose bisphosphate carboxylase‑oxygenase (RuBisCO) [9, 10]. RuBisCO is a key enzyme in photosynthesis catalysing the conversion of inorganic CO_2_ to organic carbon. It is the most abundant protein on earth, and an important model for chaperonin assisted protein folding. Since RuBisCO is an extremely inefficient catalyst, the role of CO_2_ as a greenhouse gas has drawn new attention to its role in CO_2_ conversion. RuBisCO activity has implications for crop yield, nitrogen and water usage and global carbon cycles [11]. Production of functional plant RuBisCO requires additional assembly factors that cooperate with the chaperonin [12].

The folding of *Rhodospirillum rubrum* RuBisCO (a homodimer of 51 kDa subunits) is strictly dependent on GroEL, GroES and ATP [9], but it does not require additional assembly factors. *E*. *coli* GroEL was shown to trap an unstable RuBisCO folding intermediate [10, 13]. The interaction was further characterised by mass spectrometry, which showed that RuBisCO binds to GroEL with strong negative cooperativity between the rings [14, 15]. In addition, spectroscopic studies reveal a progressive compaction of non-native RuBisCO during its interaction with GroEL and GroES, with a less compact conformation of RuBisCO in a GroEL open ring than after encapsulation under the GroES lid [16, 17].

It has been demonstrated in a number of different structural studies that folding intermediates can be trapped by rapid mixing of denatured substrate proteins with GroEL [[18], [19], [20], [21]]. In studies on GroEL complexed with non-native malate dehydrogenase (MDH), multiple conformations of unfolded MDH were observed to be bound to GroEL [20, 22]. The 3D structure of a partly folded protein, the T4 bacteriophage capsid protein gp23, was described in the folding chamber formed by GroEL and its co-chaperone gp31, a phage encoded homologue of GroES that extends the volume of the folding chamber [21]. RuBisCO in complex with GroEL-GroES has also been studied structurally, and a portion was shown to form a compact conformation that bound to both the lower part of the apical domains and the extended c-termini of GroEL [23].

In this study we use single particle cryo electron microscopy (cryo EM) to visualize folding intermediates of *R*. *rubrum* RuBisCO trapped within a GroEL ring (binary complex) at 11–12 Å resolution. In accordance with previous findings [17], the binary complex shows substrate density interacting with the apical domains of GroEL. Single particle image processing was used to sort out populations of heterogeneous and disordered assemblies. The EM density of RuBisCO shows a two-lobed density, compatible with the two domain fold of RuBisCO. The substrate density presented here may represent an early folding intermediate such as the previously reported I_1_ state of RuBisCO [13].

## 2 Results and discussion

### 2.1 Cryo-EM structure of RuBisCO intermediates bound to GroEL_D473C-6His_

**Fig. 1:**
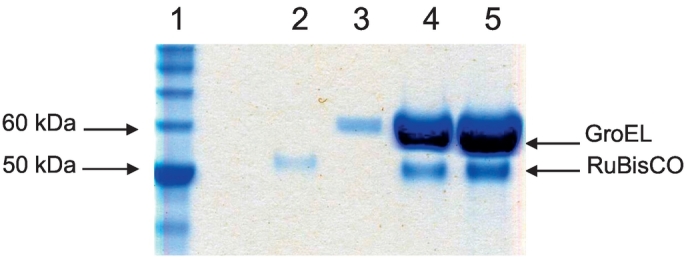
SDS PAGE of RuBisCO denatured with acid-urea and 20 mM DTT, then complexed with GroEL. Lane 1, Markers; 2, RuBisCO; 3, GroEL; 4, 7.5 μL GroEL-RuBisCO complex at 1 μM GroEL; 5, 15 μL of GroEL:RuBisCO complex.

**Fig. 2:**
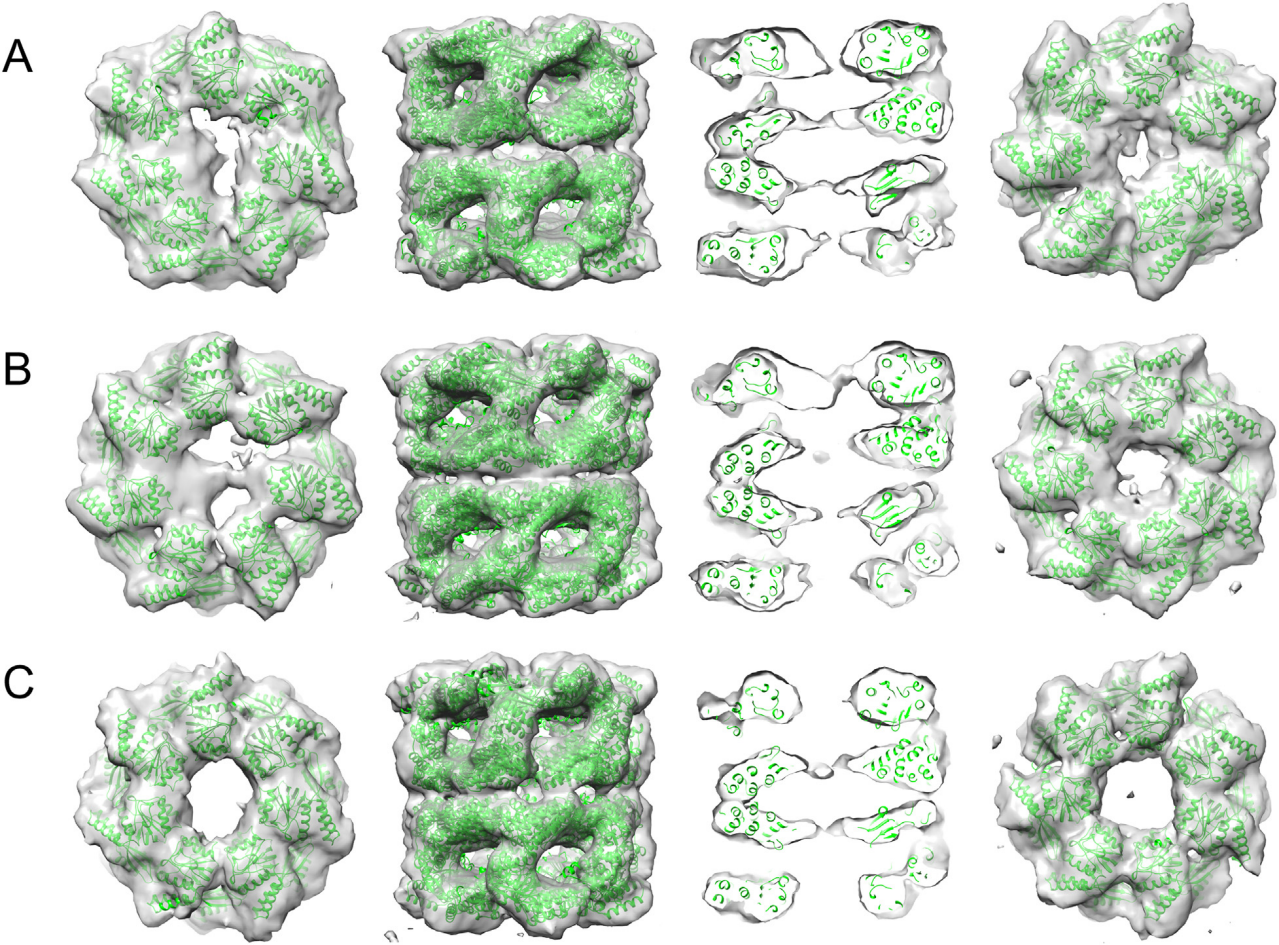
Asymmetric reconstructions of the 3 structures determined from the GroEL-RuBisCO dataset. Two classes (A, class 1, 3481 particles; B, class 2, 4845 particles) show distinct substrate density in one ring. The third class 3 (C, 6003 particles) appears empty. Each class is shown as a top view (top ring only), a side view, a central section through the side view, and a bottom view (bottom ring only). The fitted GroEL crystal structure is shown in green. The additional densities in the upper rings of A and B are attributable to bound non-native substrate. The bottom rings of A and B are about 24 Å in diameter, in comparison to ~40 Å for the empty complex (C). All maps were contoured at the 1σ level without filtering. The substrate density in A appears as disconnected features, but the presence or absence of a thin connecting region (seen in B) is likely due to the limited resolution and heterogeneity of the non-native substrate. Figure generated with Chimera [34]. (For interpretation of the references to colour in this figure legend, the reader is referred to the web version of this article.)

**Fig. 3:**
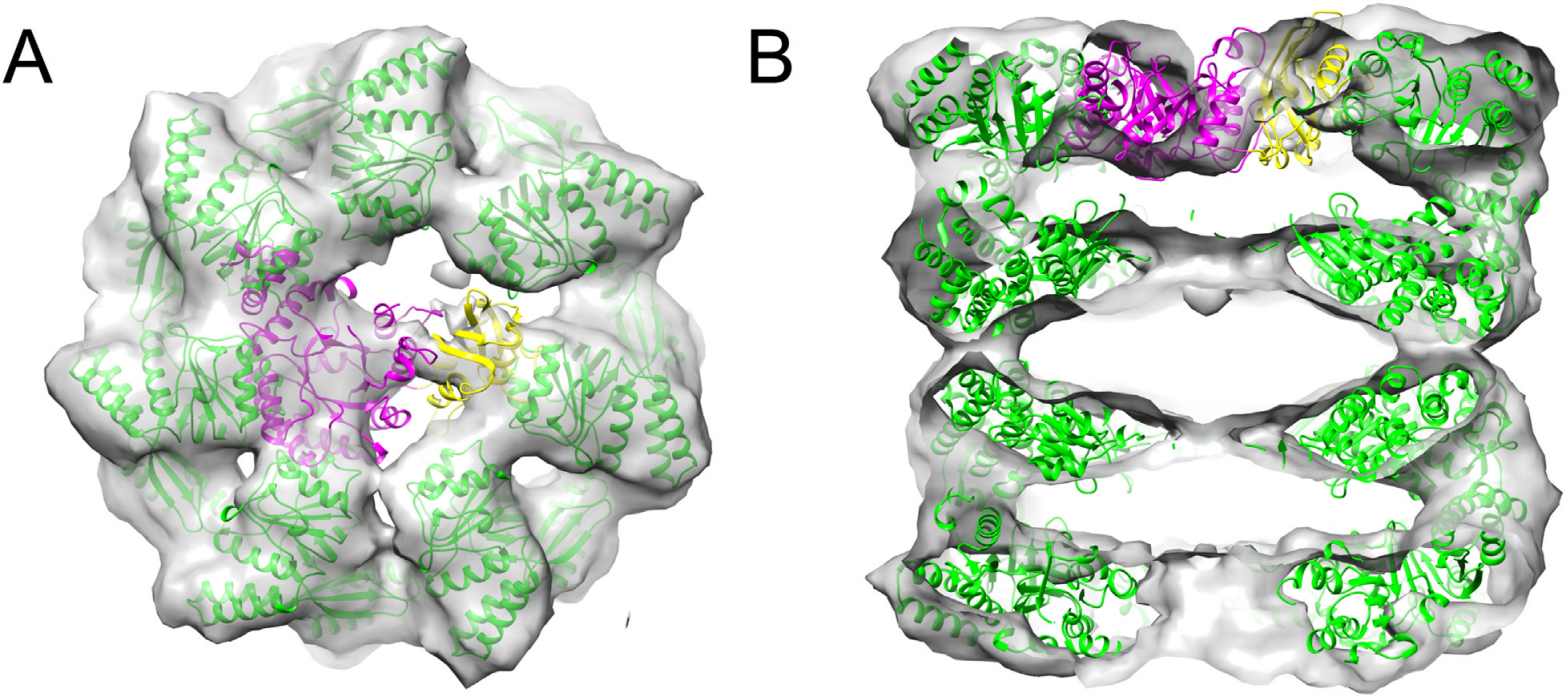
RuBisCO density from the binary complex in Fig. 2B. (A) Top view of the complex with one possible orientation of the crystal structure of *R*. *rubrum* RuBisCO A chain (PDB code 5RUB) superposed on the substrate density. The N-terminal domain (residues 1–137) is shown in yellow and the TIM barrel domain in magenta. (B) Side view section of the complex. Figure generated with Chimera. (For interpretation of the references to colour in this figure legend, the reader is referred to the web version of this article.)

GroEL-RuBisCO binary complexes were prepared by rapid dilution of RuBisCO from denaturant into GroEL-containing buffer (Fig. 1). Asymmetric reconstructions of GroEL-RuBisCO were generated after classifying a set of 15,477 particles into 3 subclasses. Two of the classes had visible substrate density and one was empty (Fig. 2A–C). The expectation based on previous GroEL-substrate complexes was that RubisCO would form disordered density located on the inner surface of the GroEL binding site [20, 21]. Remarkably, the RuBisCO density in class 2 shows a two-lobed shape (Fig. 2). A potential interpretation is that the larger substrate density represents the TIM barrel domain of RuBisCO, and the smaller substrate density is derived from the smaller N terminal domain (Fig. 3). In the two substrate occupied classes, the density attributable to RuBisCO accounts for 30–35% of the volume of the folded subunit.

Denatured RuBisCO was previously shown to form unstable, aggregation-prone folding intermediates when rapidly diluted from guanidine hydrochloride or acid neutralization [13]. The GroEL-RuBisCO binary complexes shown in this paper were formed by rapid dilution of denatured RuBisCO, so that the substrate density captured in our complexes may represent a folding intermediate trapped in a local energy minimum. Subsequent recruitment of ATP and GroES to the substrate-bound GroEL ring is likely to cause forced unfolding of the substrate protein as proposed by Lin and co-workers [17], through GroEL domain movements visualised by Clare et al. [24], giving it a fresh opportunity to escape the local energy minimum and access the global energy minimum fold.

Mass spectrometry revealed a stoichiometry of one RuBisCO per GroEL 14-mer, whereas other substrates, such as gp23 and MDH, can bind in a ratio of 2:1 [15]. The mechanism of negative cooperativity remains poorly understood. Our structures suggest a narrowing of the GroEL ring opposite to the RuBisCO bound one from ~40 Å (empty) to ~24 Å (substrate occupied) (Fig. 2, right hand column), which may be related to the negative cooperativity. A similar narrowing of the unbound GroEL trans ring was seen in the GroEL-glutamine synthetase complex [19, 25].

There is long range allosteric communication through the GroEL rings and across the ring interface. Negative cooperativity has been described both for nucleotide and for substrate binding. Some of the residues and structural elements involved in this transmission have been identified. Negative cooperativity is transmitted through the charge and van der Waal contacts in the interface between the two back-to-back rings [26, 27], possibly *via* helix D which runs from an interface contact to the nucleotide binding site or through the E461 contact. Since substrate binding affects the positions and mobility of the apical domains, this is likely to affect key inter-subunit and inter-domain salt bridge interactions that in turn affect the opposite ring.

### 2.2 RuBisCO binds to contiguous and noncontiguous sites on GroEL

Earlier *in vivo* rescue experiments on RuBisCO, using covalently linked GroEL rings (7 subunits expressed as a single polypeptide) with specific apical domain binding mutants, has shown that 3–4 adjacent, functional apical domains are required for productive folding of stringent substrate proteins such as RuBisCO and malate dehydrogenase [28]. In the substrate-bound structures presented here (class 1 and class 2, Fig. 2) the density corresponding to the RuBisCO C-terminal domain is seen in contact with 3 consecutive apical domains. The smaller density, presumed to correspond to part of the N-terminal domain, contacts an apical domain on the opposite side of the GroEL ring. The apparent domain structure of the binary complex substrate density reveals new information on the pathway of GroEL-assisted RuBisCO folding.

## 3 Materials and methods

### 3.1 Protein expression and purification

*E*. *coli* GroEL_D473C.His6_ modified to favour side views was expressed and purified with a 6-His-tag modification on the mutated cysteine 473, as described [20]. *Rhodospirullum rubrum* RuBisCO expression was carried out at 20 °C from T7 promoters in BL21 transformants in the absence of induction and were purified by ion-exchange chromatography [29, 30].

### 3.2 Sample preparation

RuBisCO (12.5 μM) was denatured by a 10-fold dilution into acid-urea (20 mM HCl, 10 M urea and 20 mM DTT). Binary complexes were then prepared by 100-fold dilution of denatured RuBisCO into 100 μL of 0.1 μM GroEL_D473C.His6_ in 50 mM HEPES pH 7.5, 10 mM KOAc, 10 mM Mg (OAc)_2_ and 5 mM DTT to a final GroEL 14-mer:RuBisCO monomer molar ratio of 1:2.5. To avoid aggregation, the addition of denatured RuBisCO was done in 3 successive steps. The mixture was incubated for 10 min at 24 °C, then centrifuged for 10 min at 13,200 rpm to remove aggregates, leaving 200 mM of residual urea. Biochemical characterisation confirmed GroEL-RuBisCo complex formation (Fig. 1). The complex was concentrated 10-fold for the SDS gel, using a 5 MWCO viva spin (Sartorius Stedim Biotech GmbH, Germany) at 13,000 rpm in a bench top centrifuge.

### 3.3 EM and image processing

3.5 μL of the complex was applied on a carbon coated C-flat grid (r2/2 grids from Protochips Inc., USA) that had been glow discharged in air for 1 min, blotted with Whatman filter paper 1 and plunge frozen in liquid ethane using a manual plunger. Images were recorded on Kodak SO-163 (Sigma UK) EM film at a magnification of 50,000 with 0.8–3.5 μm underfocus, using an FEI FEG 200 kV TEM and an Oxford CT3500 cryo holder maintained at −170 °C. The films were digitized at a step size of 7 μM using a Zeiss *Sca*i scanner, giving 1.4 Å per pixel. Defocus was determined with CTFFIND3 [31] and 160 micrographs with low astigmatism and showing Thon rings to 15 Å or beyond were selected for further processing.

Particles were extracted into 512^2^ boxes with the MRC programs XIMDISP and LABEL [32], CTF corrected, and band-pass filtered between 285 Å and 6 Å and normalized with SPIDER [33]. The boxes were then cropped and binned to 2.8 Å per pixel in 196^2^ boxes. 6157 selected side view particles were aligned to a side view from a previous 30 Å filtered EM map of empty GroEL [21]. Multivariate Statistical Analysis (MSA) in IMAGIC was used to classify the particles into 300 classes using 20 eigen images (~20 particles per class). Angular reconstitution was carried out using the class averages [34]. Two rounds of anchor set refinement were followed by one round of projection matching using SPIDER. The resulting aligned particles were classified by MSA using substrate information from eigen images showing localized variations of density in the cavities [22]. Of the three resulting classes, class1 and class 2 were occupied with RuBisCO and class 3 was empty.

Several rounds of competitive projection matching with class 2 and class 3 as reference projections yielded two classes, class 1 with RuBisCO (2771 particles) and class 2 without RuBisCO (3371 particles). Subsequently more side view particles were selected and aligned to give a working data set of 15,477 particles. Further, competitive projection matching of all 15,477 particles was carried out using preliminary C7 symmetrised 3D maps from class 1 and class 2 (occupied and Apo GroEL_D473C.His6_) each projected into 260 reference projections. After each round of projection matching and alignment, the classes were assessed for homogeneity of their constituent images by MSA. If the eigen images showed evidence of structural heterogeneity, the images were further sorted into subsets and separate 3D maps were reconstructed. Newly generated maps were reprojected as references for subsequent rounds of competitive projection matching.

The procedure of image separation was iterated a further five times with the refined models and angular steps decreasing down to 2°. At this stage, angle and class assignments were stable and MSA did not show evidence of intra-class structural heterogeneity. Of the 3 final 3D maps, two contained substrate density. Further asymmetric reconstruction was carried out for all 3 classes. For the asymmetric reconstructions, the starting model in each substrate occupied class was created by removing substrate density from the cavity such that the remaining density contacted either one or three apical domains. The choice of asymmetric starting model did not significantly affect the final asymmetric 3D reconstructions (Fig. S1). In all 3D reconstructions, maps were loosely masked to exclude noise more than 5 pixels outside the map surface. A total of 13 rounds of refinement were carried out to yield the final asymmetric map shown in Fig. 2. Class 1 and class 2 showed distinct substrate density in one ring (Fig. 2A, B) and class 3 appears empty (Fig. 2C). The map resolutions are 11–12 Å by Fourier shell correlation (Fig. S2).

GroEL PDBID:1OEL and RuBisCO domains from PDBID:5RUB were fitted as rigid bodies into the cryo-EM maps using Chimera [35].

## 4 Accession numbers

The cryo-EM maps of class 1 and 2 GroEL-RuBisCO complexes and class 3 apo GroEL are deposited in the Electron Microscopy Data Bank (EMDB) with accession codes EMD-6725–6727, respectively.

The following are the supplementary data related to this article.

Fig. S1 Reproducibility test for substrate density. Starting models for asymmetric reconstruction (A–D) were created by removing substrate density from the cavity of the class 2 binary complex, such that substrate density for only three apical domains (A), one apical domain (B) or none (C) was retained. In addition, the substrate density was completely removed from the final, asymmetric map (D). The corresponding final asymmetric 3D reconstructions are shown in E–H, with top, side view sections and bottom views of each. The choice of asymmetric starting model did not significantly affect the final reconstructions. Very similar substrate features appeared after refinement from any of the starting models, indicating an absence of reference bias. Fig. S1

Fig. S2 Fourier shell correlation curves. FSCs were calculated in Spider by dividing the dataset at the end of the reconstruction. The resolution values at 0.5 correlation are tabulated. Fig. S2

## References

[1] Balchin D., Hayer-Hartl M., Hartl F.U. (2016). In vivo aspects of protein folding and quality control. Science.

[2] Fujiwara K., Ishihama Y., Nakahigashi K., Soga T., Taguchi H. (2010). A systematic survey of in vivo obligate chaperonin-dependent substrates. EMBO J..

[3] Horwich A.L., Fenton W.A., Chapman E., Farr G.W. (2007). Two families of chaperonin: physiology and mechanism. Annu. Rev. Cell Dev. Biol..

[4] Kerner M.J., Naylor D.J., Ishihama Y., Maier T., Chang H.C., Stines A.P., Georgopoulos C., Frishman D., Hayer-Hartl M., Mann M., Hartl F.U. (2005). Proteome-wide analysis of chaperonin-dependent protein folding in *Escherichia coli*. Cell.

[5] Saibil H.R., Fenton W.A., Clare D.K., Horwich A.L. (2013). Structure and allostery of the chaperonin GroEL. J. Mol. Biol..

[6] Fayet O., Ziegelhoffer T., Georgopoulos C. (1989). The groES and groEL heat shock gene products of Escherichia coli are essential for bacterial growth at all temperatures. J. Bacteriol..

[7] Cheng M.Y., Hartl F.U., Martin J., Pollock R.A., Kalousek F., Neupert W., Hallberg E.M., Hallberg R.L., Horwich A.L. (1989). Mitochondrial heat-shock protein hsp60 is essential for assembly of proteins imported into yeast mitochondria. Nature.

[8] Ostermann J., Horwich A.L., Neupert W., Hartl F.U. (1989). Protein folding in mitochondria requires complex formation with hsp60 and ATP hydrolysis. Nature.

[9] Goloubinoff P., Christeller J.T., Gatenby A.A., Lorimer G.H. (1989). Reconstitution of active dimeric ribulose bisphosphate carboxylase from an unfoleded state depends on two chaperonin proteins and Mg-ATP. Nature.

[10] Goloubinoff P., Gatenby A.A., Lorimer G.H. (1989). GroE heat-shock proteins promote assembly of foreign prokaryotic ribulose bisphosphate carboxylase oligomers in Escherichia coli. Nature.

[11] Andersson I., Backlund A. (2008). Structure and function of Rubisco. Plant Physiol. Biochem..

[12] Bracher A., Whitney S.M., Hartl F.U., Hayer-Hartl M. (2017). Biogenesis and metabolic maintenance of Rubisco. Annu. Rev. Plant Biol..

[13] van der Vies S.M., Viitanen P.V., Gatenby A.A., Lorimer G.H., Jaenicke R. (1992). Conformational states of ribulosebisphosphate carboxylase and their interaction with chaperonin 60. Biochemistry.

[14] van Duijn E., Simmons D.A., van den Heuvel R.H., Bakkes P.J., van Heerikhuizen H., Heeren R.M., Robinson C.V., van der Vies S.M., Heck A.J. (2006). Tandem mass spectrometry of intact GroEL-substrate complexes reveals substrate-specific conformational changes in the trans ring. J. Am. Chem. Soc..

[15] van Duijn E., Heck A.J., van der Vies S.M. (2007). Inter-ring communication allows the GroEL chaperonin complex to distinguish between different substrates. Protein Sci..

[16] Lin Z., Rye H.S. (2004). Expansion and compression of a protein folding intermediate by GroEL. Mol. Cell.

[17] Lin Z., Madan D., Rye H.S. (2008). GroEL stimulates protein folding through forced unfolding. Nat. Struct. Mol. Biol..

[18] Chen S., Roseman A.M., Hunter A.S., Wood S.P., Burston S.G., Ranson N.A., Clarke A.R., Saibil H.R. (1994). Location of a folding protein and shape changes in GroEL-GroES complexes imaged by cryo-electron microscopy. Nature.

[19] Falke S., Tama F., Brooks C.L., Gogol E.P., Fisher M.T. (2005). The 13 angstroms structure of a chaperonin GroEL-protein substrate complex by cryo-electron microscopy. J. Mol. Biol..

[20] Elad N., Farr G.W., Clare D.K., Orlova E.V., Horwich A.L., Saibil H.R. (2007). Topologies of a substrate protein bound to the chaperonin GroEL. Mol. Cell.

[21] Clare D.K., Bakkes P.J., van Heerikhuizen H., van der Vies S.M., Saibil H.R. (2009). Chaperonin complex with a newly folded protein encapsulated in the folding chamber. Nature.

[22] Elad N., Clare D.K., Saibil H.R., Orlova E.V. (2008). Detection and separation of heterogeneity in molecular complexes by statistical analysis of their two-dimensional projections. J. Struct. Biol..

[23] Chen D.H., Madan D., Weaver J., Lin Z., Schroder G.F., Chiu W., Rye H.S. (2013). Visualizing GroEL/ES in the act of encapsulating a folding protein. Cell.

[24] Clare D.K., Vasishtan D., Stagg S., Quispe J., Farr G.W., Topf M., Horwich A.L., Saibil H.R. (2012). ATP-triggered conformational changes delineate substrate-binding and -folding mechanics of the GroEL chaperonin. Cell.

[25] Falke S., Fisher M.T., Gogol E.P. (2001). Structural changes in GroEL effected by binding a denatured protein substrate. J. Mol. Biol..

[26] Braig K., Otwinowski Z., Hegde R., Boisvert D.C., Joachimiak A., Horwich A.L., Sigler P.B. (1994). The crystal structure of the bacterial chaperonin GroEL at 2.8 A. Nature.

[27] Roseman A.M., Chen S., White H., Braig K., Saibil H.R. (1996). The chaperonin ATPase cycle: mechanism of allosteric switching and movements of substrate-binding domains in GroEL. Cell.

[28] Farr G.W., Furtak K., Rowland M.B., Ranson N.A., Saibil H.R., Kirchhausen T., Horwich A.L. (2000). Multivalent binding of nonnative substrate proteins by the chaperonin GroEL. Cell.

[29] Rye H.S., Burston S.G., Fenton W.A., Beechem J.M., Xu Z., Sigler P.B., Horwich A.L. (1997). Distinct actions of cis and trans ATP within the double ring of the chaperonin GroEL. Nature.

[30] Rye H.S., Roseman A.M., Chen S., Furtak K., Fenton W.A., Saibil H.R., Horwich A.L. (1999). GroEL-GroES cycling: ATP and nonnative polypeptide direct alternation of folding-active rings. Cell.

[31] Mindell J.A., Grigorieff N. (2003). Accurate determination of local defocus and specimen tilt in electron microscopy. J. Struct. Biol..

[32] Crowther R.A., Henderson R., Smith J.M. (1996). MRC image processing programs. J. Struct. Biol..

[33] Frank J., Radermacher M., Penczek P., Zhu J., Li Y., Ladjadj M., Leith A. (1996). SPIDER and WEB: processing and visualization of images in 3D electron microscopy and related fields. J. Struct. Biol..

[34] van Heel M., Gowen B., Matadeen R., Orlova E.V., Finn R., Pape T., Cohen D., Stark H., Schmidt R., Schatz M., Patwardhan A. (2000). Single-particle electron cryo-microscopy: towards atomic resolution. Q. Rev. Biophys..

[35] Pettersen E.F., Goddard T.D., Huang C.C., Couch G.S., Greenblatt D.M., Meng E.C., Ferrin T.E. (2004). UCSF chimera–a visualization system for exploratory research and analysis. J. Comput. Chem..

