# Supplementary figures and images for: A two-domain folding intermediate of RuBisCO in complex with the GroEL chaperonin
Source: Int J Biol Macromol. 2018 Oct 15;118(Pt A):671–5. doi: 10.1016/j.ijbiomac.2018.06.120 (PMC6096091; doi:10.1016/j.ijbiomac.2018.06.120)

## Slide 1
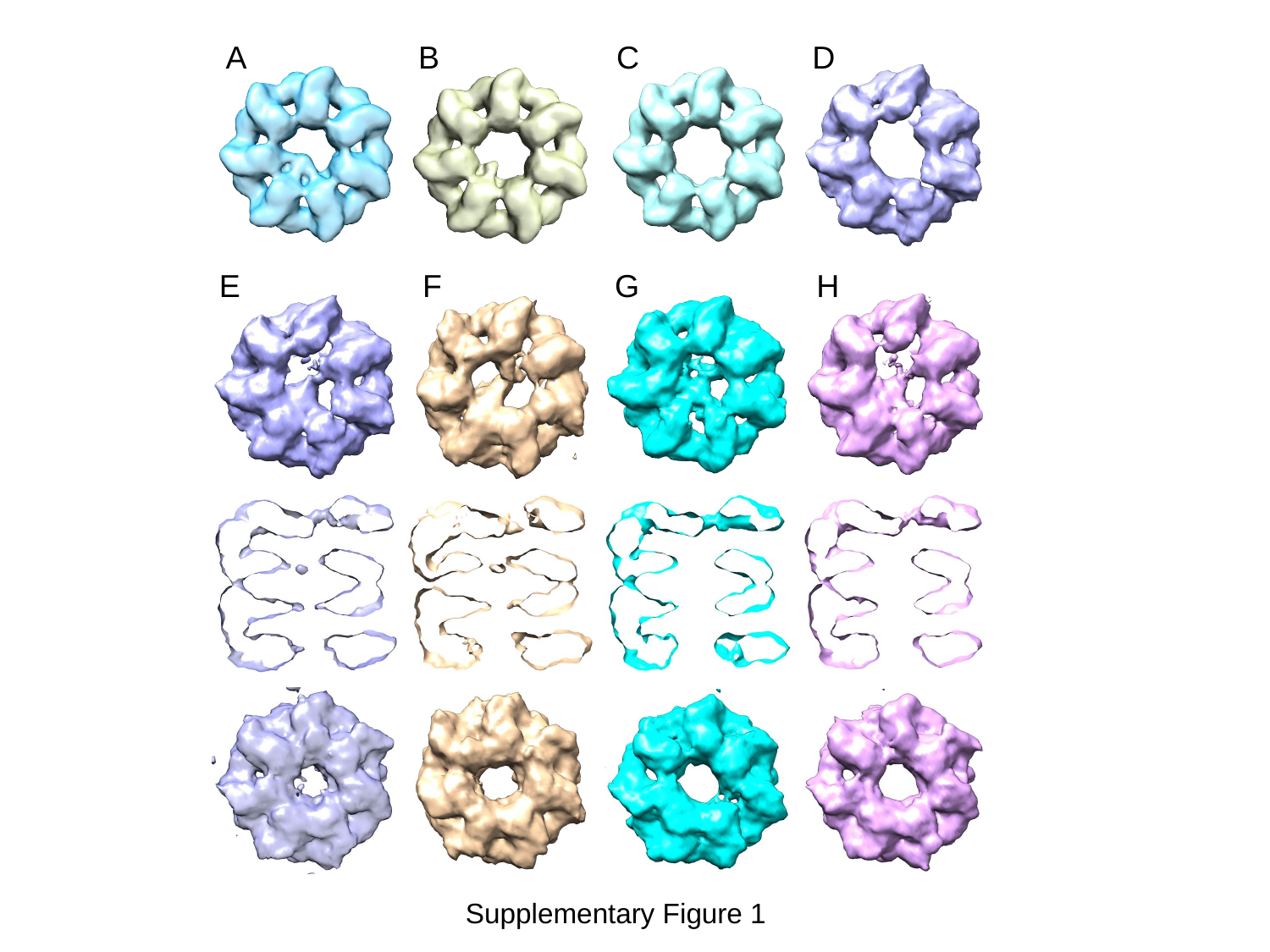

A
B
C
D
E
F
G
H
Supplementary Figure 1

Supplement: Fig. S1 — Reproducibility test for substrate density. Starting models for asymmetric reconstruction (A–D) were created by removing substrate density from the cavity of the class 2 binary complex, such that substrate density for only three apical domains (A), one apical domain (B) or none (C) was retained. In addition, the substrate density was completely removed from the final, asymmetric map (D). The corresponding final asymmetric 3D reconstructions are shown in E–H, with top, side view sections and bottom views of each. The choice of asymmetric starting model did not significantly affect the final reconstructions. Very similar substrate features appeared after refinement from any of the starting models, indicating an absence of reference bias. [file mmc1.pptx]

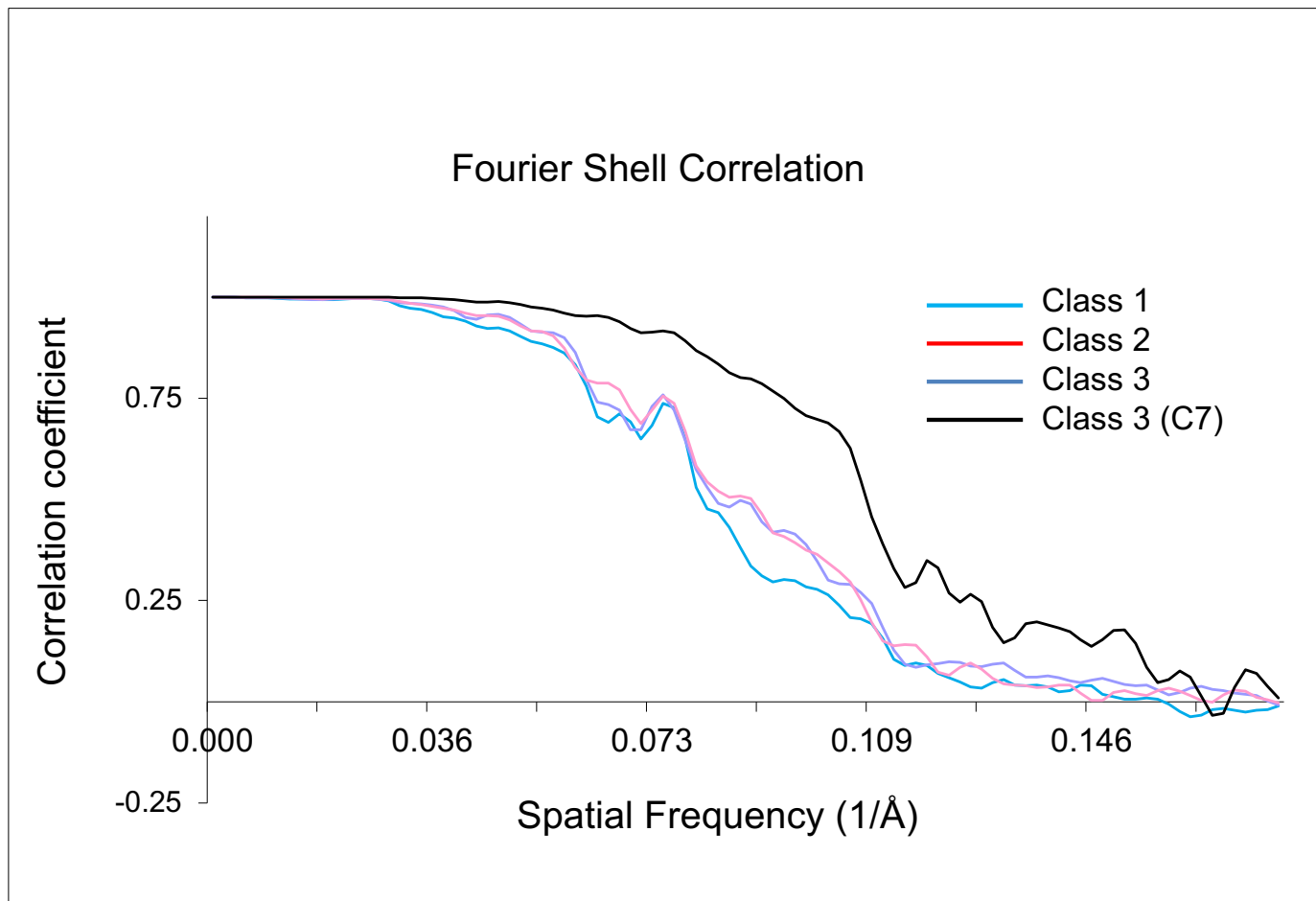

| FSC 0.5 (Å)     | C1 reconstruction | C7 reconstruction |
|-----------------|-------------------|-------------------|
| Class 1         | 12.2              |                   |
| Class 2         | 11                |                   |
| Class 3 (empty) | 11                | 9                 |

Supplementary Figure 2

Supplement: Fig. S2 — Fourier shell correlation curves. FSCs were calculated in Spider by dividing the dataset at the end of the reconstruction. The resolution values at 0.5 correlation are tabulated. [file mmc2.pdf]
